# Metacognitive beliefs, mood symptoms, and fatigue four years after stroke: An explorative study

**DOI:** 10.1371/journal.pone.0305896

**Published:** 2024-06-25

**Authors:** Synne G. Pedersen, Audny Anke, Oddgeir Friborg, Marte C. Ørbo, Mari T. Løkholm, Marit Kirkevold, Guri Heiberg, Marianne B. Halvorsen

**Affiliations:** 1 Department of Rehabilitation, University Hospital of North Norway, Tromsø, Norway; 2 Institute of Health and Society, Research Centre for Habilitation and Rehabilitation Model and Services (CHARM), Faculty of Medicine, University of Oslo, Oslo, Norway; 3 Department of Clinical Medicine, Faculty of Health Sciences, UiT–The Arctic University of Norway, Tromsø, Norway; 4 Department of Psychology, Faculty of Health Sciences, UiT–The Arctic University of Norway, Tromsø, Norway; 5 Faculty of Health Promotion, Oslo Metropolitan University, Oslo, Norway; 6 Department of Pediatric Rehabilitation, University Hospital of North Norway, Tromsø, Norway; Chinese Academy of Medical Sciences and Peking Union Medical College, CHINA

## Abstract

**Objective:**

This cross-sectional study investigated the relationship between metacognition and mood symptoms four years post-stroke and examined fatigue as a potential moderator for this relationship.

**Methods:**

A number of 143 participants completed a survey that included the Hospital Anxiety and Depression Scale (HADS), the Metacognition Questionnaire-30 (MCQ-30), the Fatigue Severity Scale (FSS), and the modified Rankin Scale (mRS) (functional status) four years after stroke. Multiple regression analyses adjusting for demographic and stroke-specific covariates were performed with anxiety and depression as dependent variables and fatigue as a moderator.

**Results:**

The proportions of participants satisfying the caseness criteria for anxiety and depression were 20% and 19%, respectively, and 35% reported severe fatigue. Analysed separately, all MCQ-30 subscales contributed significantly to anxiety, whereas only three MCQ-30 subscales contributed significantly to depression. In the adjusted analyses, the MCQ-30 subscales ‘positive beliefs’ (*p* < 0.05) and ‘uncontrollability and danger’ (*p* < 0.001), as well as fatigue (*p* < 0.001) and functional status at four years (*p* < 0.05) were significantly associated with anxiety symptoms. Similarly, the MCQ-30 subscales ‘cognitive confidence’ (*p* < 0.05) and ‘self-consciousness’ (*p* < 0.05), as well as fatigue (*p* < 0.001), stroke severity at baseline (*p* < 0.01), and functional status at four years (*p* < 0.01) were significantly associated with depression symptoms. Fatigue did not significantly moderate the relationship between any MCQ-30 subscale and HADS scores.

**Conclusion:**

Maladaptive metacognitions were associated with the mood symptoms of anxiety and depression, independent of fatigue, even after controlling for demographic and stroke-specific factors. Future studies should implement longitudinal designs to determine whether metacognitions precede anxiety or depression after a stroke, and more strongly indicate the potential of metacognitive therapy for improving the mental health of individuals after a stroke.

## Introduction

Anxiety [1], depression [2], and fatigue [3] are common in patients after a stroke. For many, these symptoms are interconnected [4] and persist beyond the first months after a stroke, significantly affecting recovery outcomes, health service utilisation, and quality of life [2, 5, 6]. However, the evidence guiding the treatment of anxiety after a stroke is limited due to the scarcity and low quality of existing studies [5]. Similarly, despite evidence supporting the use of antidepressant drugs, together with their side effects, the evidence backing the use of psychotherapy alone to treat depression after a stroke is insufficient [7]. Capobianco et al. [8] highlighted that anxiety and depression symptoms could be normal reactions after a somatic diagnosis and invasive treatments and, thus, are part of the adjustment process. Therefore, psychotherapeutic treatments focusing solely on general anxiety management skills or reality-testing of negative automatic thoughts may be inadequate since some negative beliefs or fears related to recurrent health problems are real for patients with cardiovascular disorders and stroke [8]. Accordingly, there is a pressing need for new and adapted psychological approaches to help patients with physical illnesses and emotional problems [1]. The metacognitive therapy (MCT) model may be a good candidate [9, 10] since it offers an evidence-based understanding of how general psychological beliefs (i.e. meta-beliefs) may both cause and maintain abnormal adjustment reactions and emotional symptoms. MCT can intervene in how individuals relate to their inner world of cognitions, perceptions, or emotions instead of reality-testing specific negative beliefs that may be highly relevant to the patient.

### Metacognitive therapy (MCT) and metacognition

MCT is based on a self-regulatory executive function model that describes how common cognitive processes may underlie emotional disorders across diagnoses (i.e. transdiagnostic). Metacognition refers to the way a person thinks and behaves in response to a thought, belief or feeling [10]. MCT aims to modify the cognitive processes behind maladaptive thinking styles rather than the contents of the thoughts themselves [11]. A central tenet of the metacognitive model is the cognitive attentional syndrome [10]. This is characterised by self-referential and perseverative thinking, that may include worry and rumination, threat monitoring, and use of maladaptive coping strategies [8].

Both negative and positive metacognitions drive cognitive attentional syndrome. For example, internal cues, such as dysphoric thoughts (e.g. ‘Why do I feel this way?’) may activate positive metacognitive beliefs about the usefulness of worrying or rumination to cope (e.g. ‘Worrying helps me to cope’) or negative metacognitive beliefs about worry concerning uncontrollability and danger (e.g. my worrying is dangerous for me). The rumination process is particularly important for psychological dysfunction since it perpetuates symptoms. Negative metacognitive beliefs include ideas such as ‘Worrying puts my body under a lot of stress’ and ‘Thinking this way is caused by an imbalance in my brain’. Regarding the understanding of persistent emotional distress after a stroke, the metacognitive theory suggests that dysfunctional metacognitive beliefs and emotional self-regulatory strategies (i.e. worry and rumination) impede psychological adjustment and heightens the risk of anxiety and depression after a stroke [10].

### Metacognitive beliefs & anxiety and depression

A recent meta-analytic review supported the role of metacognitive beliefs for anxiety and depression across non-clinical and mental health populations [12]. In particular, ‘negative metacognitive beliefs concerning uncontrollability and corresponding danger of worry’, as well as ‘the need for controlling thoughts’ were common across various emotional disorders. This finding aligns with recent systematic reviews focused on metacognitions in physical illnesses [8] and chronic medical conditions [13] supporting the role of negative metacognitive beliefs (‘uncontrollability and danger of worry’) for anxiety and depression. Moreover, Lenzo et al. [13] suggested that the metacognition ‘cognitive confidence’ might negatively affect patients’ coping strategies, particularly when they feel mentally fatigued.

### Post-stroke fatigue (PSF)

Long-term post-stroke fatigue (PSF) is common and present in 35%–37% of patients more than three years after a stroke [14, 15]. PSF is commonly defined as a chronic subjective feeling of lack of energy, weariness, and aversion to effort [16]. It tends to persist and has been strongly linked to negative mood symptoms [17]. Research has demonstrated associations between maladaptive metacognitive beliefs and subjective cognitive impairments in patients with chronic fatigue [18]. Cognitive and psychosocial stress can reduce the capacity for controlled cognitive processing [19] and adversely affect executive function performance [20]. Consequently, fatigue may impact the relationship between metacognitive beliefs and emotions. To our knowledge, long-term fatigue has not been investigated as a potential modifier of this relationship.

### Metacognitions post-stroke

Only one previous study has examined metacognitions and emotional symptoms after a stroke. Donnellan et al. [21] found that negative metacognitive beliefs about the ‘uncontrollability and danger of worry’ (i.e. ‘When I start worrying, I cannot stop’), ‘cognitive confidence’ (i.e., confidence in own attention and memory) and ‘cognitive self-consciousness’ (i.e. awareness of one’s thoughts) correlated with anxiety and depressive symptoms in a sample of 64 patients in the acute post-stroke phase. Metacognition remained a statistically significant covariate of anxiety and depression after adjusting for education and global cognition. This suggests that metacognitions may be relevant in the understanding of emotional symptoms post-stroke, and accordingly more research is needed to further explore and identify factors associated with abnormal adjustment reactions.

### The present study

Building on the findings of Donnellan et al. [21], we further examined the associations between maladaptive metacognitions and mood symptoms over a longer post-stroke timespan. We also question whether this relationship may be modified by concurrent fatigue. Examining how fatigue affects the relationship between metacognition and mood may provide valuable insights for any further development of MCT in somatic treatment settings to address emotional problems after a stroke.

Based on previous research, we hypothesised that negative metacognitive beliefs about the uncontrollability and danger of worry would significantly and positively predict anxiety and depression symptoms after adjusting for demographic and stroke-related factors. We do not propose specific hypotheses regarding the other metacognitions due to lack of previous studies addressing these issues among patients after a stroke [8, 13]. The study’s secondary objective was to explore whether fatigue modified the relationship between metacognitions and mood problems through interaction analyses.

## Methods

### Design and ethical approval

This cross-sectional study used questionnaire data on metacognitions, fatigue, post-stroke functioning, and anxiety and depression symptoms collected four years after a stroke. It was registered on ClinicalTrials.gov (ID: NCT03639259). The Regional Committee for Research Ethics in Medicine and Health Sciences in North Norway approved this study (institutional protocol number: 2017/1966).

### Participants

Study participants were recruited from the Norwegian arm of the ‘Rehabilitation, function and quality of life after stroke in North Norway and Central Denmark–the NorDenStroke study’. In that study, patients who experienced a verified cerebral stroke were recruited from one of three stroke units at the University Hospital of North Norway from 20 March 2014 until 31 December 2015 [22]. It excluded patients who experienced a stroke due to brain malignancy, subarachnoid haemorrhage, or brain trauma, as well as proxy responders, such as a relative who had completed a short questionnaire on behalf of a patient.

**Fig 1:**
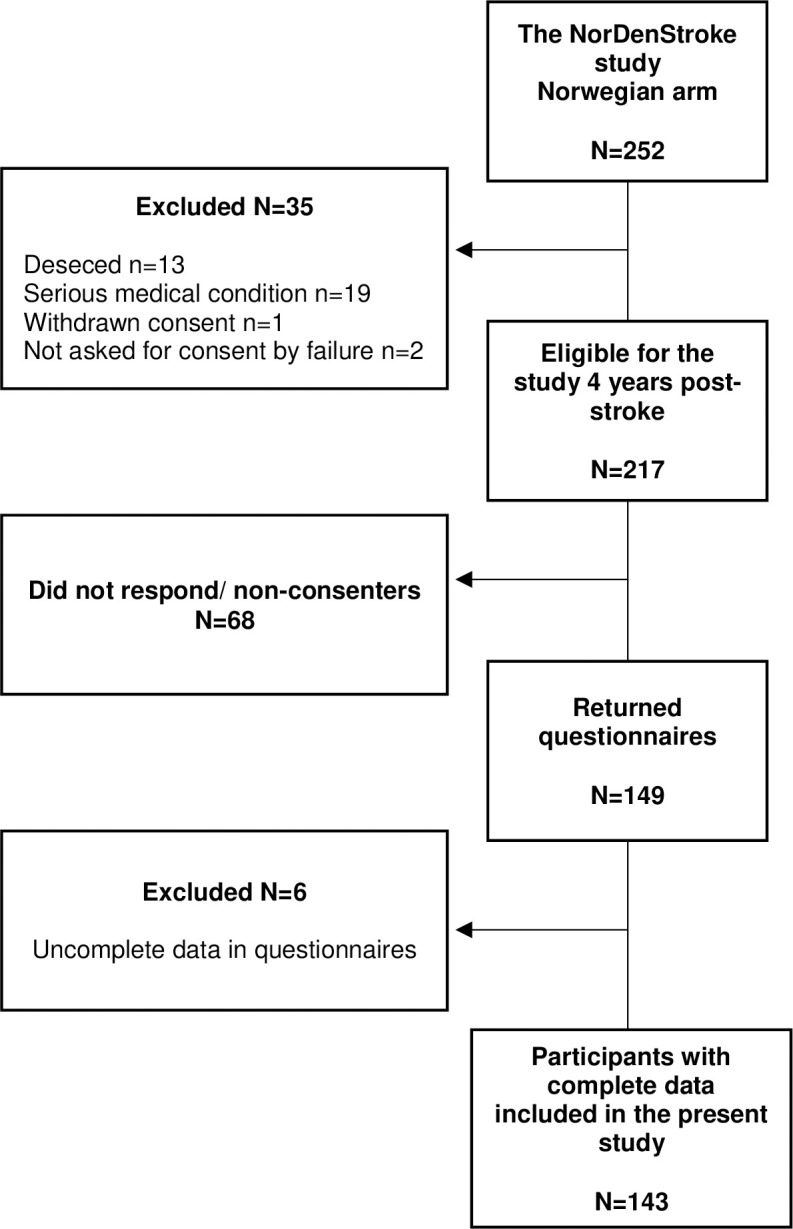
Flowchart.

The stroke survivors included in the original study met the inclusion criteria of the National Norwegian Stroke Register, clinically defined according to the World Health Organization as acute ischaemic or haemorrhagic stroke (I.63 and I.61, respectively) in individuals aged ≥18 years (International Classification of Diseases–Tenth Revision). This study, the *PostStrokeFatigue* follow-up study [14, 23], included stroke survivors who had completed questionnaires in the NorDenStroke study one year after a stroke (*n* = 217). There were no exclusion criteria for the present study. A drop-out analysis compared the 149 participants with the 68 stroke survivors who did not respond or who did not consent when invited to participate four years after their stroke (from 20 March 2018 until 31 December 2019). While the participants who did not respond or consent (*n* = 68) were significantly older than those who agreed to participate in the follow-up study (*n* = 149; mean age = 72 [standard deviation (SD) = 10.7] vs 67 [SD = 11.0] years, *p* = 0.001), they did not differ significantly in terms of sex, stroke type, or stroke severity. A flowchart following the Strengthening the Reporting of Observational Studies in Epidemiology (STROBE) guidelines is shown in Fig 1.

### Data collection procedures at four-years

A health professional contacted potential participants about this study, or information was sent to them by post. After written consent was obtained, questionnaires were sent by post. If data were missing from the questionnaire responses, participants received a follow-up telephone call and were encouraged to answer any missing questions. During this process, it was established that some participants (*n* = 5) could not complete the questionnaires due to declining health and were therefore excluded. One additional participant was excluded because of an uncompleted Metacognitions Questionnaire four years after their stroke.

### Measurements

The Hospital Anxiety and Depression Scale (HADS) measures non-vegetative symptoms of anxiety (seven items) and depression (seven items) [24]. Item scores range from 0 to 3, and the total HADS score ranges from 0 to 21; higher scores indicate increased severity. A review of 747 articles confirmed that the HADS had good validity in measuring symptom severity and clinical caseness [25]. Anxiety or depression are often clinically diagnosed based on a HADS score of ≥8 [26]. The HADS has been used in large Norwegian epidemiological studies [26, 27] and in patients after a stroke [28]. In this study, HADS’ Cronbach’s alpha values were 0.88 for anxiety and 0.81 for depression.

The Metacognitions Questionnaire-30 (MCQ-30) [29] is a short version of the original MCQ and comprises 30 items in a self-report inventory for assessing individual differences in metacognitive beliefs. Its five subscales are ‘cognitive confidence’ (e.g. ‘I do not trust my memory’), ‘positive beliefs about worry’ (e.g. ‘Worrying helps me to solve problems’), ‘cognitive self-consciousness’ (e.g. ‘I monitor my thoughts’), ‘negative metacognitive beliefs concerning uncontrollability and corresponding danger of worry’ (e.g. ‘When I start worrying I cannot stop’), and ‘beliefs about the need to control thoughts’ (e.g. ‘It is bad to think certain thoughts’). Items are scored on a four-point Likert scale from 1 (I do not agree) to 4 (I totally agree). Total subscale scores range from 6 to 24, and total MCQ-30 scores range from 30 to 120. Higher scores indicate higher levels of unhelpful metacognitions and have previously been associated with increased depressive symptoms [30]. The MCQ-30 has demonstrated good internal consistency, with Cronbach’s alpha values ranging from 0.72 to 0.93, and acceptable test-retest reliability, with Spearman’s rho values ranging from 0.59 to 0.87 [29]. In this study, the Cronbach’s alpha values for the various MCQ-30 subscales ranged from 0.76 for ‘positive beliefs about worry’ to 0.85 for ‘cognitive confidence’. For further psychometric properties of the MCQ-30, see Spada et al. [31].

The Fatigue Severity Scale (FSS) [32, 33] was used to measure participants’ fatigue four years after a stroke. The FSS has been widely used to assess fatigue in population-based stroke research [3] and has shown excellent internal consistency (Cronbach’s alpha = 0.92) and good test-retest reliability (intraclass correlation coefficient = 0.742) [34]. The FSS comprises nine items measuring fatigue in daily life across the domains of daily activities, social participation, motivation, and sleep. The FSS is graded on a seven-point Likert-type scale from 1 (no problem) to 7 (a significant problem). The Cronbach’s alpha values for the nine items ranged from 0.95 to 0.96 in this study. A global average score is calculated from the results of all nine items, with higher scores indicating greater fatigue [33]. It is recommended to define severe fatigue as an FSS score of ≥5 [33].

The modified Rankin Scale (mRS) is a six-item, global outcome scale for patients after stroke. It is used to categorise their level of functional ability and independence in activities of daily living [35]. The mRS is one of the most commonly used functional measures in stroke research and has been a primary or secondary outcome measure in recent large-scale stroke trials [36]. The mRS is a validated clinician-reported measure of global disability. However, one study showed good validity using the mRS answered by patients six months after a stroke, compared to the clinician-assessed mRS [37]. Another study reported good results using patient or proxy survey responses as an efficient and reliable alternative to generate mRS scores after subarachnoid haemorrhage compared to responses from trained personnel [38]. In this study, we provided participants with rich descriptive text for each functional category score in the mRS to help them self-evaluate.

### Data on demographic and stroke characteristics

The original study collected data on stroke-related factors and medical information from participants’ medical files. Baseline data on age, sex, stroke subtype, and stroke severity were obtained from the National Norwegian Stroke Registry.

Stroke severity was measured using the frequently used Scandinavian Stroke Scale (SSS). The advantage of the SSS is its simplicity, making repeated measures in the very acute post-stroke phase easy to perform [39]. The inter-rater reliability of its items varies from excellent for level of consciousness, orientation, and gait to moderate for facial palsy [39]. The SSS is as effective as the commonly used National Institutes of Health Stroke Scale in measuring patients’ dependency three months after a stroke [40].

Information on education, marital status, and work status was obtained from the questionnaires, whereas information on comorbidity was collected from the medical files four years after the stroke.

### Statistical analyses

The statistical analyses were conducted using SPSS Statistics software (version 28). Descriptive statistics are presented as means or medians, standard deviations (SDs), and interquartile range [IQR]). Bivariate correlational analysis used Pearson’s correlation coefficient. Differences between groups were assessed using independent samples *t*-tests for continuous variables and Chi-square tests for categorical variables.

Multivariable linear regression models are well-suited for analysing relationships between a multiple set of covariates and continuous outcome. We specified two regression models with anxiety (HADS-A) and depression (HADS-D) as dependent variables. First, each of the five MCQ-30 subscales was examined separately. The adjusted effect of each MCQ-30 subscale on HADS scores was examined by including them in separate regression analyses with the FSS and the other covariates. These analyses were structured in three blocks: the first block added the specific MCQ-30 subscale with the FSS variable; the second block added the demographics covariates (age, sex, education) and stroke-related measures (SSS severity at baseline and mRS functional status at the four-year follow-up); and the third block added the MCQ-30 subscale × FSS interaction to explore whether FSS significantly moderated the MCQ-30-HADS relationship.

In the regression analysis, the scores of all five MCQ-30 subscales were included simultaneously according to the following block specification: the first block included all five MCQ-30 subscales; the second block added the FSS; the third block added the covariates; and the fourth block added the five interaction variables (MCQ-30 × FSS) through a stepwise method. This approach ensured that only significant interactions were retained in the model. The MCQ-30, FSS, and age were treated as continuous variables and standardised to Z-scores, thus centring their part of the interaction analysis. Unstandardised beta coefficients are presented since the HADS scale range is well-known and is interpreted as the actual change in the dependent variable that occurs as a result of a unit change in the covariates. The beta coefficients are reported with confidence intervals, *P*-values, and adjusted *R*-squared values, which quantify the amount of outcome variance the predictors explain (range: 0–1).

## Results

### Participants’ characteristics

This four-year follow-up study included 143 patients who had experienced a stroke and completed the questionnaires. Their mean age at the time of injury was 67.3 years (SD = 10.9), and 60% of the participants were in the middle-age range according to the STROBE criteria (56–74 years). Most of the participants were male (64%), married (73%), and had suffered an ischaemic stroke (90%). At the time of their initial assessment, 63% were classified as mild stroke severity. Additionally, 15% of the participants held a job one year after their stroke, and 14% reported moderate to severe functional impairments in their activities of daily living four years after their stroke. Moreover, most participants (64%) had one or more comorbid conditions (Table 1). Regarding mental health outcomes, 20% met the criteria for anxiety, and 19% for depression (measured by the HADS) with threshold scores of ≥8 indicating caseness. The proportion of participants satisfying reporting severe fatigue at the four-year follow-up was 35% (Table 2).

**Table 1:** Demographic and clinical characteristics of the participants.

	Total
	*n* = 143 (100%)
Age at time of injury, mean (*SD*)	67.3 (10.9)
Age, *n* (%)	
18–55	22 (15)
56–74	85 (60)
> 75	36 (25)
Gender, *n* (%)	
Female	51 (36)
Male	92 (64)
Stroke type, *n* (%)	
Ischemic	129 (90)
Haemorrhagic	14 (10)
Scandinavian Stroke Scale (SSS), median ☯IQR]	
SSS impairment, *n* (%)	47 ☯14]
Very severe (0–14), severe (15–29) and moderate (30–44)	53 (37)
Mild (45–58)	90 (63)
4 years follow-up	
Education, n (%)	
≤10 years	54 (38)
>10 years	85 (59)
Missing data	4 (3)
Marital status, *n* (%)	
Married/cohabitant	105 (73)
Widowed/single	38 (27)
Work status, *n* (%)	
Working	21 (15)
Retired/sick leave/unemployed	122 (85)
Modified Rankin Scale, *n* (%)	
No symptoms/ no significant functional impairment/ slight functional impairment (0–2)	123 (86)
Moderate or severe functional impairment (3–5)	20 (14)
Comorbidity, *n* (%)	
No comorbidity	52 (36)
Comorbidity, one additional condition	63 (44)
Multimorbidity, two or more additional conditions	28 (20)

Comorbidity: Heart disease (included hypertension) *n* = 74; cancer *n* = 23; intestinal disease *n* = 3; metabolic disease *n* = 6; migraine *n* = 5; epilepsy *n* = 6; rheumatism *n* = 9

**Table 2:** Prevalence of fatigue and mood symptoms.

	*n* (%)
FSS scores	
<5	93 (65)
≥5	50 (35)
Non-fatigue	67 (47)
Borderline-fatigue (>4<5)	26 (18)
High-fatigue	50 (35)
HADS-A	
<8	115 (80)
≥8	28 (20)
No case (0–7)	115 (80)
Possible case (8–10)	15 (11)
Probable case (11–21)	13 (9)
HADS-D	
<8	116 (81)
≥8	27 (19)
No case (0–7)	116 (81)
Possible case (8–10)	17 (12)
Probable case (11–21)	10 (7)

Abbreviations: FSS: Fatigue Severity Scale, HADS-A: Hospital Anxiety and Depression Scale-Anxiety Symptoms, HADS-D: Hospital Anxiety and Depression Scale-Depression Symptoms

### Bivariate correlations and group comparisons

Table 3 presents the bivariate correlations between participants’ MCQ-30 subscale, FSS, and HADS scores and their characteristics four years after a stroke. The MCQ-30 subscale ‘need to control thoughts’ was significantly associated with age (*p* < 0.05) and education (*p* < 0.001). Comparison between groups defined based on the covariates showed that older participants had lower education levels (*p* = 0.001) and a greater need to control thoughts (*p* = 0.03). Sex was significantly associated with mRS scores (*p* < 0.05), FSS scores (*p* < 0.001), MCQ subscale ‘uncontrollability and danger’ scores (*p* < 0.01), HADS anxiety scores (*p* < 0.001), and HADS depression scores (*p* < 0.01). In the group comparisons, women had higher mRS scores (worse functional status) (*p* = 0.014) and higher FSS scores (more severe fatigue) (*p* < 0.001) than men four years after their stroke. All the MCQ-30 subscales correlated with anxiety symptoms, and all but ‘self-consciousness’ correlated significantly with depression (Table 3).

**Table 3:** Pearson correlations between MCQ-30 subscales, FSS, and HADS scores and participant characteristics (*n* = 143) four years after stroke.

Variable	Mean (SD)	1	2	3	4	5	6	7	8	9	10	11	12	13
1. Age	70.9 (11)	-												
2. Gender		-.02	-											
3. Education		-.27^a^	.07	-										
4. Stroke severity scale		.07	.15	-.10	-									
5. Modified ranking scale		.10	-.21^c^	-.12	-.19^c^	-								
6. Fatigue severity scale	3.98 (1.8)	.03	.32^a^	-.07	-.11	.26^b^	-							
7. MCQ-cognitive confidence	10.8 (4.35)	.02	-.10	-.14	-.00	.11	.22^b^	-						
8. MCQ-positive beliefs	9.31 (3.65)	.10	-.13	-.27^a^	-.06	.04	.26^b^	.47^a^	-					
9. MCQ-self-consciousness	12.29 (3.95)	.12	-.10	-.11	-.05	.03	.09	.19^c^	.54^a^	-				
10. MCQ-uncontrollability	9.94 (3.85)	-.03	.22^b^	-.18^c^	.01	.09	.28^a^	.41^a^	.70^a^	.49^a^	-			
11. MCQ-need to control thoughts	10.55 (3.87)	.21^c^	-.04	-.27^a^	-.02	.08	.21^b^	.37^a^	.64^a^	.65^a^	.55^a^	-		
12. HADS-A	4.45 (3.98)	-.13	-.32^a^	-.09	-.07	.25^b^	.48^a^	.29^a^	.49^a^	.21^b^	.61^a^	.25^b^	-	
13. HADS-D	3.84 (3.57)	-.01	.22^b^	-.06	-.24^b^	.43^a^	.51^a^	.34^a^	.37^a^	.08	.33^a^	.25^b^	.63^a^	-

Abbreviations: MCQ-30 = metacognitive questionnaire -30, HADS = hospital anxiety and depression scale, A = anxiety, D = depression, SD = standard deviation

^a^ *p* ≤ .001

^b^ *p* ≤ .01

^c^ *p* ≤ .05

### Multivariable linear regression models

The regression models were specified with anxiety (HADS-A) and depression (HADS-D) as dependent variables, respectively. When the MCQ-30 subscales were examined separately, all contributed significantly to anxiety, whereas only three (‘positive beliefs’, ‘uncontrollability and danger’, and ‘cognitive confidence’) contributed significantly to depression. Fatigue did not significantly moderate the relationship between any MCQ-30 subscale and HADS scores, regardless of whether the covariates were excluded or included (Table 4).

**Table 4:** Multiple regression analyses examining MCQ subscales as predictors of anxiety and depression at follow-up (four years after stroke).

	Anxiety symptoms HADS-A (range 0–14)					Depression symptoms HADS-D (range 0–14)				
Variables	*R* ^2^	Crude beta	Adj ^1^ beta _95%CI_	Int ^1^ beta	FSS ^1^ beta _95%CI_	*R* ^2^	Crude beta	Adj ^1^ beta _95%CI_	Int ^1^ beta	FSS ^1^ beta _95%CI_
**MCQ subscales** (one-by-one)										
Positive beliefs	0.428	1.56^a^	**1.67**^**a**^_1.12 | 2.22_	0.08	1.23^a^_0.65 | 1.80_	0.438	0.78^a^	**0.81**^**a**^_0.34 | 1.29_	-0.09	1.52^a^_1.02 | 2.02_
Uncontrollability/danger	0.498	2.04^a^	**1.98**^**a**^_1.47 | 2.49_	-0.14	1.20^a^_0.67 | 1.73_	0.421	0.61^c^	**0.65**^**b**^_0.17 | 1.12_	-0.28	1.59^a^_1.09 | 2.09_
Cognitive confidence	0.296	0.77^c^	**0.65**^**c**^_0.07 | 1.23_	0.01	1.51^a^_0.89 | 2.14_	0.443	0.88^a^	**0.81**^**a**^_0.36 | 1.26_	-0.28	1.59^a^_1.10 | 2.08_
Need to control thoughts	0.301	0.63^c^	**0.75**^**c**^_0.14 | 1.35_	-0.33	1.49^a^_0.86 | 2.12_	0.389	0.03	-0.01−_0.47 | 0.46_	0.04	1.75^a^_1.25 | 2.25_
Self-consciousness	0.301	0.65^c^	**0.70**^**c**^_0.13 | 1.27_	0.14	1.60^a^_0.99 | 2.22_	0.389	0.03	-0.01−_0.47 | 0.46_	0.04	1.75^a^_1.25 | 2.25_
1: **MCQ subscales** (all included)	0.395					0.185				
Positive beliefs		0.80^c^	**0.96**^**c**^_0.18 | 1.73_				0.58	0.62−_0.09 | 1.33_		
Uncontrollability/danger		2.01^a^	**1.86**^**a**^_1.14 | 2.57_				0.17	0.25−_0.40 | 0.90_		
Cognitive confidence		-0.01	-0.16−_0.70 | 0.39_				0.60^c^	**0.50**^**c**^_0.01 | 1.00_		
Need to control thoughts		-0.74^c^	-0.62−_1.36 | 0.12_				0.25	0.26−_0.41 | 0.94_		
Self-consciousness		-0.23	-0.25−_0.91 | 0.41_				-0.62^c^	**-0.67**^**c**^_-1.27 | -0.08_		
2: **FSS** (fatigue)	0.502	1.33^a^	**1.17**^**a**^_0.64 | 1.71_			0.400	1.65^a^	**1.41**^**a**^_0.92 | 1.91_		
3: **Covariates**	0.516					0.466				
Age (Z-score)			-0.48−_0.99 | 0.03_					-0.09−_0.55 | 0.37_		
Gender (0_female_, 1_male_)			-0.63−_1.70 | 0.45_					-0.01−_0.99 | 0.97_		
Education (0_≤10 yrs_, 1_>10 yrs_)			0.11−_0.95 | 1.18_					0.21−_0.75 | 1.17_		
SSS baseline (0_mild_, 1_moderate-severe_)			-0.17−_1.19 | 0.85_					**1.27**^**b**^_0.33 | 2.20_		
mRS (0_minor_, 1_moderate-severe_)			**1.52**^**c**^_0.08 | 2.97_					**2.02**^**b**^_0.64 | 3.40_		
4: **Interactions** (fatigue)			ns					ns		

*Notes*. Statistically significant MCQ subscales (*p* < .05) are marked as bold. Upper part of table: MCQ subscales examined separately one at a time. Lower part of table: MCQ subscales examined simultaneously, all included in one analysis. Block numbers (1: 2: 3: and 4:) tell the sequence of variable inclusions. Crude beta = The effect of MCQ adjusted for FSS only, Adj beta = The effect of MCQ adjusted for FSS plus all covariates

^1^ Adjusted age, gender, education, SSS and mRS. Int beta = Interactions (MCQ subscale*FSS) were explored in the last block in all analyses. ns = Fatigue did not significantly moderate the MCQ–HADS relationship in any interaction tests.

^a^ *p*-value < .001

^b^ *p*-value < .01

^c^ *p*-value < .05

^95% CI^ 95% confidence interval. *R*^2^ = Adjusted R-square. Intercept final model = 4.70 (anxiety) and 2.93 (depression). MCQ = Metacognitions Questionnaire, FSS = Fatigue Severity Scale, SSS = Scandinavian Stroke Scale (stroke severity), mRS = modified Rankin Scale (functional status after stroke).

In the combined model including all MCQ-30 subscales for HADS-A, the ‘positive beliefs’ (*beta* = 0.96, *p*<0.05) and ‘uncontrollability and danger’ (*beta* = 1.86, *p*<0.001) subscales, as well as FFS (*beta* = 1.17, *p*<0.001) and mRS (*beta* = 1.52, *p*<0.05) were significant predictors of anxiety symptoms. The final model with covariates included in block three explained 51% of the variance in anxiety symptoms, of which 39.5% of the variance was accounted for by the measured metacognitions.

Similarly, in the combined model that included all MCQ-30 subscales for HADS-D, the subscales ‘cognitive confidence’ (*beta* = 0.50, *p*<0.05) and ‘self-consciousness’ (*beta* = −0.67, *p*<0.05), as well as FFS (*beta* = 1.41, *p*<0.001), stroke severity at baseline (*beta* = 1.27, *p*<0.01), and mRS four years after a stroke (*beta* = 2.02, *p*<0.01) were significant predictors of depression symptoms. The main effect model incorporating covariates explained 46% of the variance in depression symptoms, of which 18.5% of the variance was accounted for by the measured metacognitions. The role of metacognitions thus seems more prominent for anxiety than for depression.

In the final block, we explored whether fatigue moderated the relationship between any of the five MCQ subscales and either anxiety or depression. However, none of these interaction tests were significant. Notably, the relatively large discrepancies between the unadjusted and adjusted *beta*-weights are due to the strong correlation between some of the MCQ-30 subscales (Table 3), resulting in a sizeable downward adjustment that turned some of the weakest positive associations into negative ones.

## Discussion

This study’s findings contribute new knowledge about the association of maladaptive metacognition with anxiety and depression symptoms in the context of patients who had a stroke and whether differences in fatigue levels moderate this relationship. To our knowledge, this study is the first to explore this relationship in the long-term post-stroke phase.

Our results partially align with our initial hypothesis by showing that the maladaptive metacognitive ‘negative beliefs concerning uncontrollability and danger of worry’ was associated with long-term symptoms of anxiety but not depression four years after a stroke. This association persisted even after accounting for fatigue and adjusting for demographic and stroke-related factors. Interestingly, our findings diverge from Donnellan et al. [21], who found that this exact metacognitive domain was associated with both anxiety and depression symptoms. Two recent systematic reviews [8, 13] on patients with cancer or epilepsy reported a similar association.

The MCQ-30 subscale ‘positive beliefs about worry’ was also associated with anxiety symptoms and persisted after adjusting for the same covariates. Similarly, the MCQ-30 subscales ‘cognitive confidence’ (i.e. lack of confidence in memory) and ‘self-consciousness’ (i.e. persistent attention to one’s thought processes) were both associated with depressive symptoms after adjusting for covariates. These findings align closely with those of Donnellan et al. [21], reinforcing the robustness and consistency of the observed associations across different studies within the context of post-stroke metacognitive influences on anxiety and depression symptoms.

Compared to our study, Donnellan et al. [21] had a smaller sample size (*n* = 64) and focused on patients during the acute post-stroke phase. Despite these disparities in sample size and the timing of assessments, both studies observed numerous maladaptive metacognitions associated with mental health, spanning the acute and long-term post-stroke phases, highlighting the potential significance of examining maladaptive metacognition to understand the persistence of abnormal adjustment reactions, such as anxiety and depression. This consistency across studies reinforces the notion that maladaptive metacognitive processes may play a pivotal role in the enduring impact on mental health after a stroke, regardless of the stage at which the study cohort is examined.

Our findings revealed that PSF did not moderate the relationship between maladaptive metacognition and anxiety and depression symptoms in the later post-stroke phases. However, it must be noted that fatigue was prevalent within our cohort and emerged as a significant predictor for both anxiety and depression symptoms. To our knowledge, this is the first study that explore how PSF may influence the relationship between metacognitions and mood symptoms after a stroke. Nonetheless, the optimal way to measure PSF remains uncertain, indicating the need for future studies on stroke, mood disorders, and metacognition. The lack of interaction effects in our study holds practical significance. It suggests that all patients with mood disorders after a stroke could benefit from MCT, irrespective of their fatigue levels.

However, fatigue might significantly moderate the relationship between metacognition and mood in earlier post-stroke stages. Another potential moderating factor worth exploring in future studies but not addressed in our study is cognitive control of executive functions [41], as Donnellan et al. highlighted [21]. Future studies examining the interplay between mood and metacognitive beliefs in patients after a stroke should focus on assessing post-stroke cognitive health, particularly executive functions, based on both performance-based and subjective reports [21, 40].

The outcomes of our exploratory study, conducted in the long-term aftermath of a stroke, align with the MCT model [9, 10]. This model suggests that maladaptive metacognition may underpin abnormal emotional adjustment reactions through self-focused attention (i.e. the cognitive attentional syndrome) consisting of perseverative negative thinking, threat monitoring, and behaviours that prevent the experience of being able to tolerate thoughts and feelings without having to deal with them in any particular way. Our findings substantiate the initial observations made by Donnellan et al. [21] and underscore the need for further research into the relationship between metacognitions and mood among patients after a stroke. Such research would enhance our understanding of the psychological factors contributing to the development and persistence of emotional problems after a stroke. Therefore, MCT strategies hold promise as potential interventions for preventing and addressing emotional sequelae after a stroke.

### Strengths and limitations

One strength of our study is its complete questionnaire data for all participants. Self-reported data can provide a wide range of responses and is valuable for obtaining the individual’s perspectives and opinions. However, self-reported measures can be subject to response biases and may not fully capture the constructs of interest. One limitation of our study was its cross-sectional design, with data collected only at one time point after a stroke, which limits the ability to draw causal inferences about the relationships between metacognitive beliefs, mood symptoms, and fatigue. Future studies should employ longitudinal design to substantiate causal relationships more strongly. The hypothesis that fatigue would significantly moderate the relationship between metacognitive beliefs and mood symptoms failed in the present study despite a good sample size for a clinical study. Further research should therefore explore other moderators or methodological approaches for probing other modifying factors, as for example other psychological or cognitive factors, such as cognitive control or executive function. Another limitation of our study was the lack of a control group, introducing uncertainty about whether the observed relationships are unique to the stroke population or if they might also be present in the general population or in other patient groups. A further limitation of our study is that the participants had strokes of mild and moderate strokes severity, thus limiting the generalisability of our findings to this patient subgroup. Hence, an important next step in future studies would be a randomized controlled trial comparing cognitive behavioural therapy with metacognitive therapy, with the inclusion of more severe strokes as well as a healthy control group.

## Conclusions

This study highlights the enduring relationship between metacognitive beliefs, mood symptoms, and fatigue. To our knowledge, this is the largest study on the relationship between metacognitions and mood symptoms in a stroke population and the first to investigate PSF as a potential moderator of the relationship between the MCQ-30 and HADS. Its findings align with existing research suggesting the potential efficacy of the MCT approach in addressing emotional challenges in patients with somatic or neurological conditions. Specifically, they underscore the relevance of metacognitive beliefs in understanding the co-occurrence of emotional disorders following a stroke.

While this study refrained from establishing causal relationships, it is evident that more robustly designed studies are needed to clarify these associations. There is a pressing need for more effective treatments for anxiety and depression after a stroke. Notably, metacognitive interventions have shown promise for improving mental health among patients with other physical illnesses, underscoring their potential applicability for patients after a stroke.
